# Metformin Induces Changes in Sphingosine-1-Phosphate-Related Signaling in Diabetic Mice Brain

**DOI:** 10.3390/ijms26199832

**Published:** 2025-10-09

**Authors:** Przemysław Leonard Wencel, Kinga Czubowicz, Magdalena Gewartowska, Małgorzata Frontczak-Baniewicz, Robert Piotr Strosznajder

**Affiliations:** 1 Laboratory of Preclinical Research and Environmental Agents, Mossakowski Medical Research Institute, Polish Academy of Sciences, 02-106 Warsaw, Poland; kczubowicz@imdik.pan.pl (K.C.); rstrosznajder@imdik.pan.pl (R.P.S.); 2 Electron Microscopy Research Unit, Mossakowski Medical Research Institute, Polish Academy of Sciences, 02-106 Warsaw, Poland; mgewartowska@imdik.pan.pl (M.G.); mbaniewicz@imdik.pan.pl (M.F.-B.)

**Keywords:** diabetes, sphingosine-1-phosphate, sphingosine kinases, metformin, inflammation, hippocampus, ultrastructure

## Abstract

Type 2 diabetes mellitus (T2DM) is a chronic disease that has become a serious health problem worldwide. Moreover, increased systemic and cerebrovascular inflammation is one of the major pathophysiological features of T2DM, and a growing body of evidence emphasizes T2DM with memory and executive function decline. Bioactive sphingolipids regulate a cell’s survival, inflammatory response, as well as glucose and insulin signaling/metabolism. Moreover, current research on the role of sphingosine kinases (SPHKs) and sphingosine-1-phosphate receptors (S1PRs) in T2DM is not fully understood, and the results obtained often differ. The aim of the present study was to evaluate the effect of metformin (anti-diabetic agent, MET) on the brain’s sphingosine-1-phosphate-related signaling and ultrastructure in diabetic mice. Our results revealed elevated mRNA levels of genes encoding sphingosine kinase 2 (SPHK2) and sphingosine-1-phosphate receptor 3 (S1PR3), which was accompanied by downregulation of sphingosine-1-phosphate receptor 1 (S1PR1) in the hippocampus of diabetic mice. Simultaneously, upregulation of genes encoding pro-inflammatory cytokines interleukin 6 (IL-6) and tumor necrosis factor α (TNF-α) was observed. Administration of MET significantly reversed changes in mRNA levels in the hippocampus and reduced *Sphk2*, *Il6,* and *Tnf*, with concomitant upregulation of *S1pr1* gene expression. Ultrastructural analysis of diabetic mice hippocampus revealed morphological alterations in neurons, neuropil, and capillaries that were manifested as mitochondria swelling, blurred synaptic structure, and thickened basal membrane of capillaries. The use of MET partially reversed those changes. Our research emphasizes the important role of insulin sensitivity modulation by metformin in the regulation of SPHKs and S1PRs and inflammatory gene expression in a murine model of T2DM.

## 1. Introduction

The global prevalence of type 2 diabetes (T2DM) is increasing rapidly. The data from epidemiological studies demonstrate that in 2021, over 537 million people were diagnosed with diabetes, with increasing numbers diagnosed at younger ages [1]. Moreover, estimates imply that people with diabetes die, on average, 5–14 years earlier (depending on the time when T2DM was diagnosed) than people without diabetes [1]. T2DM is a chronic multifactor disease that is characterized by disturbance in carbohydrate and fat metabolism and results in abnormal persistent hyperglycemia. It may be due to insulin resistance (brain, muscle, adipocyte, or hepatic), progressive β-cell failure, and apoptosis. In patients with T2DM, chronic hyperglycemia and metabolic aberrations are gradually responsible for causing damage to various organs, leading to the development of many diabetic complications, including microvascular complications (neuropathy, retinopathy, nephropathy) that cause a considerable burden for patients and the healthcare system. Diabetes also increases the risk of developing vascular dementia (relative risk (RR = 2.48)), Alzheimer`s Disease (RR = 1.46), and mild cognitive impairment (RR = 1.21) [2]. In T2DM, hyperglycemia leads to the activation of astrocytes and increased production of pro-inflammatory cytokines, which initiates an inflammatory cascade in the central nervous system (CNS) and inevitably leads to neuro-inflammation and cerebrovascular damage [3]. Impairment of the hippocampal function and cognitive deficits have been observed in many animal obesity and T2DM models. Some of these observations reveal several functional and structural changes (including a reduction in microvessels) in the CA1 and dentate gyrus regions of the hippocampus, as well as a reduction in synaptic plasticity, and impairment of long-term potentiation and spatial memory [4,5,6,7]. Similar observations are present in the hypothalamus, which is considered to be the most vulnerable to high-fat diet (HFD)-induced neurocognitive issues evoked by increased cytokine production, neuronal stress, reactive oxygen species, and insulin resistance [8]. In addition to the hippocampus and hypothalamus, the brain cortex and cerebellum have also been revealed as vulnerable to the detrimental effects of HFD as well as T2DM. Many studies showed that HFD decreases hippocampal neurogenesis, reduces synaptic density and plasticity, induces mitochondrial dysfunction, astrocyte activation, and induces neurodegeneration damage, which result in impairments in learning and memory, locomotor functioning, and anxiety-like behavior [9,10,11,12,13,14].

Consumption of HFD leads to an excess of free fatty acid that promotes dysregulation of glucose homeostasis and targets insulin-sensitive tissues, including the brain [15]. Ceramides are members of the bioactive sphingolipid (BS) family and a major cellular lipid bilayer membrane component. They are also a product of free fatty acid metabolism that accumulates in various types of cells and leads to lipotoxicity in many metabolic disorders, including diabetes and obesity [16]. Bioactive sphingolipids, such as ceramide and sphingosine-1-phosphate (S1P), are crucial molecules that regulate biological processes, from programmed cell death and senescence to cell survival and proliferation. Moreover, BS modulates the inflammatory response and could serve as a potential target for diagnosing and treating various neuro-inflammatory and metabolic diseases. [17,18]. S1P is synthesized by phosphorylation of sphingosine (Sph) using sphingosine kinases (SPHKs), which is produced via a salvage pathway by the hydrolysis of complex sphingolipids and ceramide. SPHKs are important cell proliferation, migration, survival, and apoptosis regulators. They are also essential in neural and vascular development. Mice that do not express *Sphk1* or *Sphk2* are viable, but deletion of both genes at the same time is lethal [19]. Even though both kinases are responsible for the phosphorylation of sphingosine to S1P, their function and cellular localization differ. SPHK1 is mainly present in the cytosol, while SPHK2 is located in the nucleus, endoplasmic reticulum, and mitochondria. SPHK2 can also shuttle between the nucleus and cytoplasm upon phosphorylation that is catalyzed by protein kinase D and may modulate functions such as cell proliferation and survival [20]. In contrast to SPHK2, SPHK1 contributes more to the level of S1P in circulation, and SPHK2 is the predominant active isoform in the liver, kidney, pancreatic islets, and brain [21]. Many studies demonstrate that sphingolipid metabolites, including ceramide and S1P, are involved in β-cell apoptosis and play an important role in the pathology of T2DM [21,22,23,24].

Metformin (MET) is a biguanide drug that has been widely used in Europe since 1957 (as well as in the USA since its FDA approval in 1994) as a first-line therapy for newly diagnosed T2DM patients to treat hyperglycemia. MET effectiveness as an anti-diabetic drug started to be recognized worldwide after the publication of study results of the UK Prospective Diabetes Study Group, where the effect of intensive blood–glucose control with MET on complications in overweight patients with T2DM was presented. Moreover, in the same study, the tendency toward a reduced risk of microvascular endpoints was observed [25]. Metformin exerts its anti-diabetic effect mainly by suppression of hepatic gluconeogenesis and by enhancing insulin suppression of endogenous glucose production. Additionally, MET can stimulate AMP-activated protein kinase, a key regulator of lipid metabolism and oxidative/endoplasmic reticulum stress [26,27]. Metformin exerts pleiotropic effects, as many research studies suggest that the drug acts on multiple tissues through various underlying mechanisms rather than on a single organ via a unifying mode of action [28]. Even though MET was invented a long time ago, the exact molecular mechanisms of its therapeutic action in T2DM remain poorly understood [26]. Moreover, there is limited knowledge about the influence of MET on S1P signaling, especially in the brain. MET has also been shown to cross the blood–brain barrier and reach the CNS [29]. Besides anti-diabetic activity, MET also has other therapeutic properties, including anti-inflammatory, immunomodulatory, antitumor, and protective effects on the brain, liver, and pancreatic tissues [30,31].

Since the MET mechanism of action and its effect on brain S1P levels exposed to T2DM are poorly understood, we investigated its effect on the expression of several genes encoding the following: sphingosine kinases, S1P receptors, and pro-inflammatory cytokines in the hippocampus of diabetic mice. Moreover, ultrastructural changes in animals receiving a high-fat diet with streptozotocin (STZ), as well as the response to the metformin, were assessed.

## 2. Results

### 2.1. Model of Diabetes

**Figure 1:**
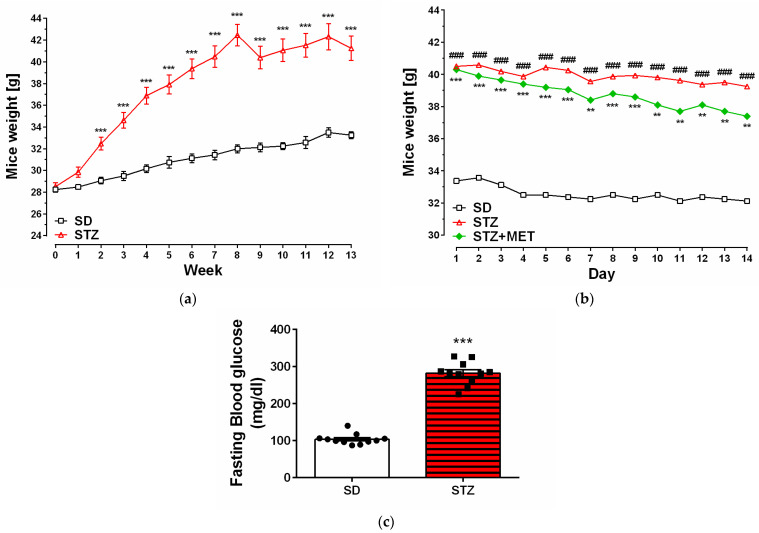
Animal weight [g] during experiment (**a**); MET administration (**b**); and blood glucose levels of control (SD) and diabtetic (STZ) mice [mg/dL] (**c**). ** *p* < 0.01; ***, ### *p* < 0.001 compared to the SD animals (8–16 for each group); ANOVA with Tukey’s post hoc test.

After two weeks of HFD consumption, STZ animals started to have significantly higher body weight than animals from the control group (SD) that received standard chow diet (Figure 1a). Administration of MET slightly reduced body weight of STZ + MET animals (Figure 1b). Diabetic mice had 2.8 times higher blood glucose levels compared to the control animals receiving standard feed (Figure 1c).

### 2.2. Sphingosine Kinases, S1P Receptors and Pro-Inflammatory Cytokine Expression in Hippocampus of Diabetic Mice

**Figure 2:**
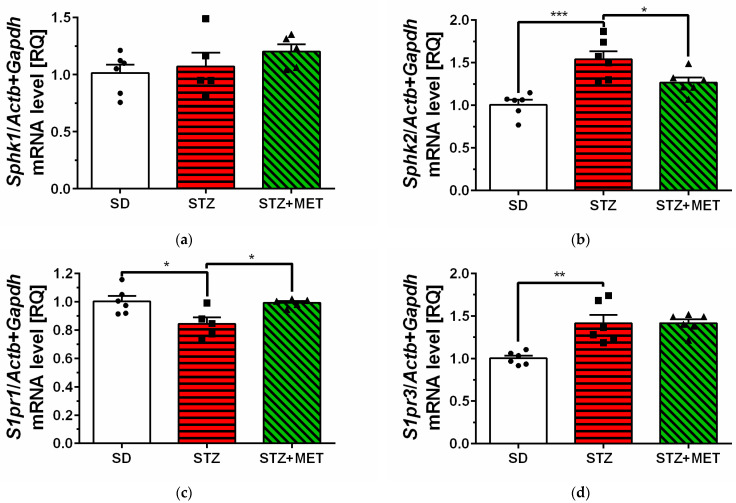
Changes in the mRNA levels of sphingosine kinase 1 (**a**) and −2 (**b**) (*Sphk1* and *−2*), and receptors of sphingosine-1-phosphate (*S1pr1* (**c**) and *−3* (**d**)) measured using real-time PCR in the brain hippocampus of control animals on a standard diet (SD), animals received a high-fat diet and streptozotocin (STZ) and mice were simultaneously treated with metformin (STZ + MET). * *p* < 0.05; ** *p* < 0.01; *** *p* < 0.001 as compared to the appropriate controls (5–6 animals for each group); ANOVA with Tukey’s post hoc test.

**Figure 3:**
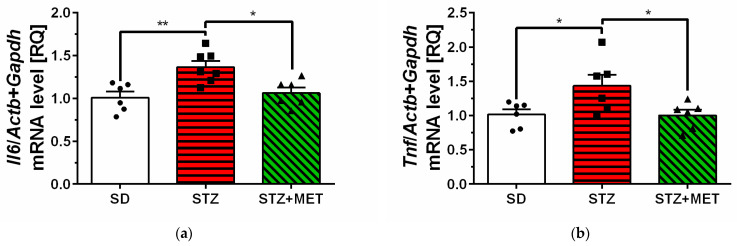
Changes in the mRNA levels of pro-inflammatory cytokines *Il6* (**a**) and *Tnf* (**b**), measured using real-time PCR in the brain hippocampus of control animals on a standard diet (SD), animals received a high-fat diet and streptozotocin (STZ), and mice were simultaneously treated with metformin (STZ + MET). * *p* < 0.05; ** *p* < 0.01 as compared to the appropriate controls (6–7 animals for each group); ANOVA with Tukey’s post hoc test.

The mean expression levels of genes encoding sphingosine kinase 2 (*Sphk2*) and sphingosine-1-phosphate receptor 3 (*S1pr3*) were significantly higher in the hippocampus of STZ animals compared to control animals (SD) (Figure 2b,d). Similarly, upregulation of SPHK2 protein levels was observed in STZ mice brain, but no significant changes were observed in S1PR1 and −3 protein levels (Supplementary Figure S1a–c). In the hippocampus of diabetic mice, we have observed significant reduction in sphingosine-1-phosphate receptor 1 (*S1pr1*) mRNA levels (Figure 2c). We also observed significant upregulation of genes encoding pro-inflammatory cytokines interleukin 6 (*Il6*) and tumor necrosis factor α (*Tnf*) in the hippocampus of STZ mice (Figure 3a,b). Metformin administration significantly reversed changes in mRNA levels in the hippocampus and reduced *Sphk2*, *Il6,* and *Tnf* mRNA levels, with concomitant upregulation of *S1pr1* gene expression (Figure 2b,c and Figure 3a,b).

### 2.3. Ultrastructure of Diabetic Mice Hippocampus

**Figure 4:**
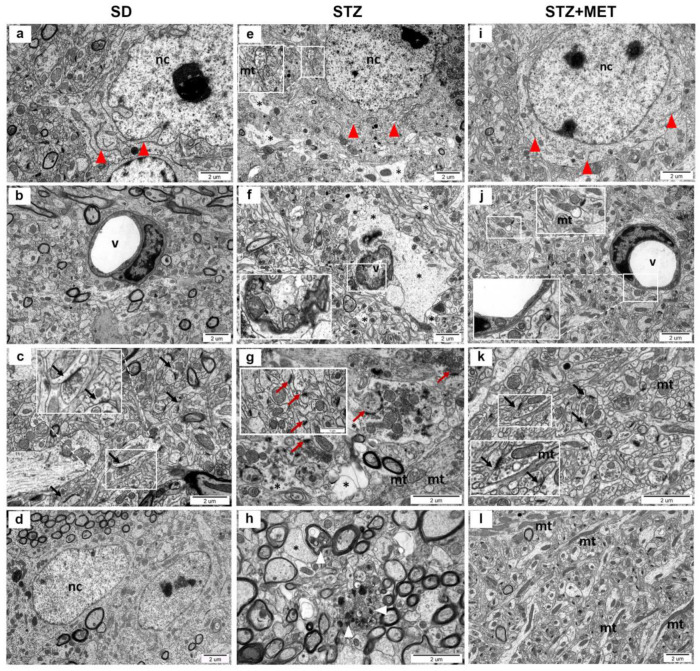
Ultrastructural alterations in the murine hippocampus. Representative electron micrographs of hippocampal tissue from the following: SD, control animals (**a**–**d**); STZ, animals with experimentally induced diabetes (**e**–**h**); STZ + MET diabetic animals treated with metformin (**i**–**l**). Labeled structures: neuronal cells (nc), blood vessels (v), endoplasmic reticulum (red arrowheads), mitochondria (mt), ultrastructurally intact synapses (black arrows), ultrastructurally altered synapses (red arrows), regions of swelling (*), and neurodegenerative features (white arrowheads).

Ultrastructural analysis of hippocampal tissue from mice with induced diabetes revealed profound morphological alterations affecting neurons, neuropil, and capillaries (as compared to the control animals presented in Figure 4a–d). Neuronal cells exhibited dilated endoplasmic reticulum cisternae (Figure 4e), while some mitochondria showed degenerative changes, including swelling, loss of cristae, and disruption of the outer membrane (Figure 4e, insert). The neuropil frequently displayed signs of edema (Figure 4e,f). Synaptic pathology was also apparent, with notable swelling of presynaptic terminals, irregular distribution and accumulation of synaptic vesicles (Figure 4g), and, in some instances, blurred synaptic architecture. In some nerve endings, a reduction in the number of synaptic vesicles was observed, with some synapses completely devoid of vesicles (Figure 4g, insert). Capillaries in the diabetic hippocampus displayed thickened basal membranes (Figure 4f), accompanied by pronounced edema of perivascular astrocytic end-feet and a narrowing of the vascular lumen. Additionally, neurodegenerative features, such as electron-dense inclusions and membrane disintegration, were present in neuronal terminals (Figure 4h). In contrast, the hippocampal ultrastructure in diabetic mice treated with MET was largely preserved. Mitochondrial alterations were limited to isolated cases, showing partial loss or blurring of cristae (Figure 4j,k, inserts). The ultrastructure of the capillary wall was maintained, although mild swelling of perivascular astrocytic processes was still evident (Figure 4j). Notably, an increased number of elongated mitochondria was observed within neuronal terminals of the neuropil (Figure 4l).

## 3. Discussion

A growing body of evidence emphasizes the role of SPHKs/S1P in diabetes, and their effect on the progression of the disease. In the present work, we have observed a significant upregulation of the gene encoding SPHK2 in the hippocampus of diabetic mice. Similar to our observation, Mastrocola et al. observed elevated *Sphk2* mRNA levels and no change in *Sphk1* mRNA levels in the livers of db/db rats. These alterations were accompanied by elevated SPHK1 and −2 as well as S1PR1 protein levels [32]. SPHK2 appears to be an important enzyme in the pathogenesis of T2DM but its role in the diabetic brain is rather scarce [21]. It has been observed that high glucose exposure to pancreatic beta cells increases S1P levels by activation of SPHK2. Moreover, inhibition of SPHK activity by use of SKI in C57BL6 mice and mouse insulinoma-6 (MIN6) cell line impaired glucose tolerance and decreased plasma insulin levels, which suggest the crucial role of SPHKs and S1P in glucose secretion [23]. A novel study revealed that the expression of enzymes involved in bioactive sphingolipid metabolism (especially polymorphism of the *Sphk2* gene) may contribute to the genetic predisposition to type 1 diabetes, and these gene polymorphisms are correlated with the degree of cellular islet autoimmunity [33]. A study characterizing Sphk2 ^−/−^ mice revealed that they are protected from age-related obesity and metabolic decline, which may be in part due to elevated adiponectin levels and enhanced lipolysis in adipose tissue [34]. Moreover, SPHK2 deficiency in HFD + STZ mice significantly prevented the loss of β-cell mass, preserved insulin production, and ameliorated the diabetic phenotype [22]. A relatively recent study found that neither S1P levels nor S1P/Sph ratio is associated with the risk of diabetes. However, at the same time, dhS1P (dihydro-S1P) and dhS1P/dhSph (dihydrosphingosine) ratios are elevated in serum up to five years before diabetes onset, suggesting that SPHK2 activity could be stably associated with T2DM predisposition. Interestingly, global deletion of *Sphk1* in mice results in a more than 50% reduction in S1P in serum, whereas loss of *Sphk2* unexpectedly increases circulating S1P [35]. While the pro-inflammatory role of SPHK1 is quite well described, the results from studies on SPHK2 are contradictory and depend on disease models. It is suggested that the pro-apoptotic effect of SPHK2 is independent of catalytic activity and depends on its translocation from the nucleus to the cytoplasm, as observed by the redistribution of SPHK2 poll in the nucleus and cytoplasm after treatment of β-cells with palmitic acid [21,22]. The deletion of Sphk2−/− in the mice leads to an increase in anti-inflammatory (M2) macrophage levels in obstructed kidneys and a decrease in the mRNA levels of genes encoding the following inflammatory cytokines: MCP1, TNF-α, CXCL1, and IL-1β [36].

The effectiveness of MET on glucose production, glucose tolerance, and insulin sensitivity in T2DM has been demonstrated in numerous research studies [37,38,39]. Potential modulatory effects of MET on bioactive sphingolipid levels have been demonstrated in many diabetic- and non-diabetic-associated diseases. Metformin was effective in reducing multiple ceramide concentrations and modulating S1P levels [40,41,42]. Moreover, administration of MET to the HFD-rats successfully reduced the expression of genes encoding enzymes involved in the synthesis of ceramide in the kidney, as well as reversed the elevated expression of the gene encoding SPHK2 [41]. Our study showed that the administration of MET significantly reduced upregulated mRNA levels of the gene encoding SPHK2, whereas it had no effect on SPHK1 in the hippocampus of HFD + STZ mice. Several studies have demonstrated the downregulatory effect of MET on SPHK1 activity and expression mostly in the periphery, but the data about its effect on SPHK2 expression have not been extensively studied, especially in the brain [43]. Additionally, recent data suggest that SPHKs are potent targets for MET therapy in cancer treatment [44,45].

Among all S1P receptors, S1PR1 has the highest expression level in the CNS and is involved in the regulation of neurons and synaptic actions. Loss of S1PR1 regulatory capabilities may lead to altered immune cell trafficking and vascular endothelial barrier function and some studies suggest that biased agonists of S1PR1 may achieve vascular protective effects in diabetes and metabolic and cardiovascular diseases [46,47,48]. In the present study, we have observed significant downregulation of the gene encoding S1PR1. These results correspond with our previous research, where the reduction in the gene encoding S1PR1 was observed in the brain cortex and hippocampus of obese mice [11]. Skoug et al. recently revealed that in insulin-resistant rats, despite higher levels of S1P in plasma, the cortical levels remained comparable to the control animals, and this effect was accompanied by lower density of S1PR1 and S1PR4 in nerve-terminal-enriched membranes. On the contrary, in the same study, obese HFD-fed mice had elevated plasma and cortical concentrations of S1P, with a decreased density of S1PR1 and S1PR4 [49]. A study by Silva et al. showed that S1PR1 levels are reduced in the hypothalamus of HFD-fed rats or mice and leptin-deficient ob/ob mice, suggesting its role in regulating food intake [50]. These findings suggest the important role of S1P signaling in T2DM-associated synaptic dysfunction. Additionally, in our study, the use of MET reversed downregulated *S1pr1* mRNA levels in the hippocampus to the control values, thus suggesting its neuroprotective effect. Pro-survival and anti-apoptotic action of MET resulting from MET-S1PR1 binding have been shown previously, where MET was demonstrated to protect neurons against sevoflurane-induced apoptosis through activating the S1PR1-dependent ERK1/2 signaling pathway [51]. While S1PR1 is responsible for vascularization and neurogenesis during development, S1PR3, which is highly expressed in astrocytes and microglia, is involved in gliosis and neuro-inflammation processes [19,52,53]. Most vascular endothelial cells are now known to express S1PR1 and S1PR3, whereas stimulation of S1PR1 leads to angiogenesis, and activation of S1PR3 may lead to the impairment of barrier function [54]. In the present work, we observed that the expression of S1PR3 was significantly higher in the hippocampus of STZ animals compared to control animals. Chakrabarty et al. revealed that mRNA levels of S1PR3 are increased in the adipose tissue of HFD-induced obese mice [55]. Interestingly, loss of this receptor in combination with HFD resulted in impairment of adiposity, glucose homeostasis, and inflammation.

Growing evidence suggests microvascular dysfunction as one of the key underlying mechanisms responsible for this state, and cerebral microvascular dysfunction is observed in pre-diabetic patients, indicating that microvascular disease processes occur before the onset of diabetes [56,57]. Microvascular dysfunction that occurs during T2DM has a remarkable impact on brain functioning and is related to increased inflammatory and immune response, oxidative stress, and blood–brain barrier permeability [57]. In our diabetic mice, we have observed numerous alterations in the morphology of hippocampal capillaries, which were manifested as a thickened basal membrane. In a study using a similar model (HFD + STZ) but using rats, diabetes induced femoral artery ultrastructural damage, which was accompanied by elevated levels of TNF-α, AGEs (advanced glycation end products), ET-1 (endothelin−1), and iNOS (inducible nitric oxide synthase) [58]. The administration of MET to the HFD + STZ rats substantially protected the femoral artery ultrastructure by improving endothelial cell morphology, including the nucleus and plasma membrane, forming the intimal surface and intact external elastic lamina [58]. Another study has found that cardiac capillaries of rats with diabetes mellitus (DM) have significantly higher density, their arrangement was more disordered, and their surfaces were highly irregular. Moreover, microvessels showed increased permeability and significant destruction of the microvascular wall. All those changes were accompanied by the deregulation of S1PR1 and S1PR3 protein and gene expression, suggesting their involvement in microvascular complications in diabetes [59].

In the present work, we have observed significant upregulation of pro-inflammatory mRNAs of interleukin 6 (*Il6*) and tumor necrosis factor α (*Tnf)* in the hippocampus of HFD + STZ mice. Those alterations were accompanied by many pathological changes observed during ultrastructural analysis. Elevated levels of pro-inflammatory cytokines in the hippocampus have been strongly associated with hippocampal-dependent memory impairments, as we previously reported in animals after HFD, and in the works of other authors [11,14]. Upregulation of inflammatory markers closely linked to endothelial cell injury (IL-1β, IL-6, TNF-α, and TGF-β1) in the hippocampus and memory deficits has also been recently observed in HFD + STZ mice [60]. Even short-term consumption of HFD impairs synaptic plasticity and long-term memory in the aged hippocampus, and it does so via elevations in IL-1β. The receptor blockade with the IL-1 receptor antagonist in aged HFD-fed rats rescues late phase long-term potentiation [12]. Moreover, HFD consumption induces pro-inflammatory cytokines TNF-α and IL-6, which directly correlate with increased permeability of the blood–brain barrier in the hippocampus, deficits in memory and mood behavior, and finally, impairment in mitochondrial function and astrocyte activation, resulting in hippocampal dysfunction [14]. One of the most important effects of MET in diabetes is its anti-inflammatory effect. Our study demonstrated that the administration of MET significantly downregulated elevated expression of *Il6* and *Tnf* genes in HFD + STZ mice. In a study using mice primary hepatic cells treated with TNF-α, the use of MET strongly inhibited TNF-α-dependent expression of pro-inflammatory cytokines [61]. The anti-inflammatory effect of MET was also observed previously in HFD mice, where its administration reduced the expression of *Il1β*, *Il6*, *Ifn-γ*, *Gfap*, *Iba1,* and *CD68* in the hippocampus as well as *Il1β*, *Il6*, *Tnf*, *Mcp-1*, *Gfap*, and *CD68* in the pre-frontal cortex [31]. In this study, we did not assess the behavioral parameters in our model following MET treatment; however, beneficial effects of MET on the hippocampus and behavior of diabetic animals have been previously described. Notably, a two-week administration of MET can effectively restore the isoflurane- and STZ-induced hippocampal tissue damage, cognitive and memory impairment as well as downregulate inflammatory factors involved in the NF-κB signaling pathway [62].

Ultrastructural analysis of T2DM brains of mice used in this study revealed significant morphological alterations in neurons, where mitochondria exhibited signs of degeneration, including swelling and disruption of the outer membrane. Structural abnormalities were also present in synapses, where swelling of the presynaptic compartment, and, in some cases, a blurred synaptic structure were observed. Furthermore, features indicative of neurodegeneration were present within neuronal terminals. These alterations in neuronal morphology correspond with another study using a similar model where HFD + STZ mice had irregularly arranged neurons with hyperchromatic nuclei and pyknosis. In contrast, control animals showed neatly arranged hippocampal neurons [63]. In our study, the administration of MET slightly improved those changes; however, we also noted mild swelling of perivascular astrocytic end-feet. Similar results were observed in diabetic rats’ hypothalamus, where most mitochondria have damaged structural organization, which was associated with both swelling of the matrix and destruction of cristae. Supplementation of rats with MET normalized neuronal architecture almost to the control stage, improved all altered parameters, and re-established mitochondrial structure [64]. Moreover, Natrus et al. revealed that neurons of T2DM rats showed signs of apoptosis, partially fragmented and enlarged tubules of rough ER, accumulation of fragmented mitochondria that underwent the process of autophagy and formed mitophagosomes, pyknotically altered nuclei with signs of chromatin condensation, and nuclei fragmentation [65]. Additionally, the authors observed swelling of synaptic terminals with loss of vesicles with neurotransmitters in the neuropil. The administration of MET slightly improved the morphological signs of damage in the nervous tissue and caused better preservation of the mitochondrial structure [65]. These results indicate that metformin can effectively improve diabetic brain ultrastructure and may modulate the expression of genes encoding proteins involved in inflammatory response. Even though many novel anti-diabetic drugs have appeared on the market, MET, thanks to its pleiotropic effects and good safety profile, still offers a promising target for the treatment of many metabolic and non-metabolic diseases.

## 4. Materials and Methods

**Figure 5:**
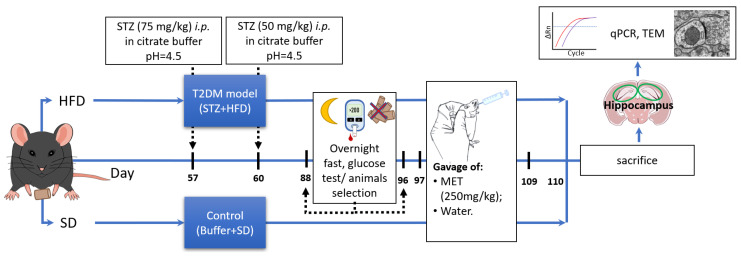
Schematic representation of the diabetic mouse model used in this study.

All experiments were approved by the II Local Ethics Committee for Animal Experimentation in Warsaw (approval no. WAW2/065/2019) and carried out following the ARRIVE guidelines and the EU Directive 2010/63/EU. Male C57BL/6J mice (10–12 weeks, 28 ± 2 g) from The Animal House of the Mossakowski Medical Research Institute PAS, Warsaw, Poland, were given high-fat diets (HFD (Ssniff Spezialdiäten GmbH, Soest, Germany): 60 kJ% Fat, 20 kJ% Protein, 20% kJ Carbohydrates, E15742-34, corresponding to Research Diets, Inc. D12492) for 16 weeks. The control group received a standard chow diet (SD (Ssniff Spezialdiäten GmbH, Soest, Germany): 9 kJ% Fat, 24 kJ% Protein, 67% kJ Carbohydrates). All groups of mice were housed individually in plastic breeding cages in an air-conditioned room with mechanical ventilation, a regulated 12 h dark–light cycle, a temperature of 20–24 °C and humidity of 55% (+/−10%). In all cages, environmental enrichments in the form of cotton rolls and wooden fibers for building a nest were applied. At the beginning of the experiment, no statistically significant body weight differences existed between both experimental groups. To create the T2DM model, HFD mice, after 8 weeks on the high-fat diet, were treated with 75 mg/kg streptozotocin, *i.p.* (Sigma-Aldrich, St Louis, MO, USA), followed 3 d later with a second dose of streptozotocin (50 mg/kg). SD mice received appropriate control (0.1 M citrate buffer, pH = 4.5). On week 12 on the HFD, blood glucose tests were performed after an overnight (14–16 h) fast. Mice with blood glucose ≥ 11.1 mM (200 mg/dl) that was measured 4 weeks later were considered diabetic. After 96 days on HFD, the animals started receiving metformin (MET) (oral gavage, 250 mg/kg b.w., Cayman Chemical Company, Ann Arbor, Michigan, USA) in water daily for 2 weeks while the control animals received vehicle (water). One day after the last MET administration, the animals were euthanized by decapitation. The brain hippocampus was quickly isolated on an ice-cold glass Petri dish, and samples were immediately frozen in liquid nitrogen and stored at −80 °C for qPCR analysis (Figure 5).

### 4.1. Glucose Test

A measurement of glucose levels was performed in mice after 12 weeks on HFD. The animals fasted overnight for 16 h (6:00 PM–10:00 AM) before the glucose test. Blood glucose levels were measured from the tail vein with an Accu-Check Performa glucometer (Roche Diabetes Care GmbH, Mannheim, Germany).

### 4.2. Gene Expression Analysis

The brain hippocampus was isolated on ice and flash-frozen in liquid nitrogen. RNA was extracted using TRI-reagent, according to the manufacturer’s protocols (Sigma-Aldrich, St Louis, MO, USA). DNA was digested with DNase I (Sigma-Aldrich, St Louis, MO, USA). The concentration and purity of RNA were assessed spectrophotometrically (A260/A280 method). Reverse transcription of 4 μg of total RNA was performed with avian myeloblastosis virus reverse transcriptase and random primers (High Capacity Reverse Transcription Kit, Thermo Fisher Scientific Baltics UAB, Vilnius, Lithuania). Real-time PCR was performed on Applied Biosystems 7500 Real-Time PCR System (Thermo Fisher Scientific, Inc., Waltham, MA, USA) using TaqMan Fast Advanced Master Mix, following the manufacturer’s guidelines and using specific mouse primers (Applied Biosystems, Life Technologies Corporation life technologies corporation, Pleasanton, CA, USA): *Sphk1* (Mm00448841_g1), *Sphk2* (Mm00445021_m1) *S1pr1* (Mm02619656_s1), *S1pr3* (Mm02620181_s1), *Il6* (Mm00446190_m1), *Tnf* (Mm00443258_m1). Each sample was analyzed in three to four replicates. Gene expression was calculated using the ΔΔCt method, and normalized against beta-actin (*Actb*, Mm00607939_s1) and (*Gapdh*, Mm99999915_g1), and the results were expressed as relative quantities (RQs).

### 4.3. Immunochemical Determination of Protein Levels (Western Blot Analysis)

Immunochemical analysis of protein level was performed using the Western blotting method in standard conditions. Tissue samples were homogenized in T-PER™ Tissue Protein Extraction Reagent (Thermo Fisher Scientific Inc., Rockford, IL, USA), determination of protein level using the BCA method was performed and then the samples were mixed with Laemmli buffer, and denatured at 95 °C for 5 min. After standard SDS-PAGE separation, the proteins were “wet”-transferred to nitrocellulose membranes in standard conditions and used for immunochemical analysis with specific antibodies, followed by chemiluminescent detection. The membranes were washed for 5 min in TBST (Tris-buffered saline with Tween 20 buffer: 100 mM Tris, 140 mM NaCl and 0.1% Tween 20, pH 7.6) and non-specific binding was blocked for 1 h at room temperature (RT) with 5% non-fat milk solution in TBST. Membranes were probed with the following primary antibodies: Sphk2 (1:500, 5% BSA, overnight 4 °C, Protein Tech, Rosemont, IL, USA), S1PR1 (1:500, 5% BSA, overnight 4 °C, Sigma-Aldrich, St Louis, MO, USA), S1PR3 (1:500, 5% BSA, overnight 4 °C, Invitrogen). The membranes were washed three times in TBST, incubated for 60 min at RT with appropriate secondary antibodies (1:2000 anti-rabbit, IgG, Sigma-Aldrich, St Louis, MO, USA), and washed again three times in TBST. Antibodies were detected using chemiluminescent reaction and ECL reagent (Bio-Rad Laboratories, Hercules, CA, USA, Clarity Western ECL Substrate) under standard conditions. After each protein detection, the membranes were stripped (25 mM Glycine-HCl, 1% (*w*/*v*) SDS, pH 2; 30 min at room temperature) and re-probed. The GAPDH (1:40,000, Sigma-Aldrich, St Louis, MO, USA) was used as a loading control for molecular-weight proteins. In all experiments, densitometry analysis of immunoblots was performed using normalization to the immunoreactivity of GAPDH. Densitometric analysis and size-marker-based verification were performed with TotalLab v1.11 software (NonLinear Dynamics Ltd., Newcastle upon Tyne, UK).

### 4.4. Ultrastructural Analysis of Hippocampus by TEM

The mice were anesthetized with a ketamine–xylazine (KX) cocktail in a dose of 50/5 mg/kg b.w., respectively, and perfused first with 0.9% NaCl in 0.01 M sodium–potassium phosphate buffer (pH 7.4), and then with 2% paraformaldehyde and 2.5% glutaraldehyde in 0.1 M cacodylate buffer (pH 7.4). The material was sequentially fixed in 2% paraformaldehyde, 2.5% glutaraldehyde in cacodylate buffer, and post-fixed in 1% osmium tetraoxide with 0.8% potassium ferricyanide for 2 h. Hippocampus were dehydrated in a series of ethanol gradients, embedded in epoxy resin, and polymerized at 60 °C for 24 h. The ultra-thin sections were stained with uranyl acetate and lead citrate. Images were acquired using a JEM-1011 EX (Jeol, Tokyo, Japan) transmission electron microscope equipped with a MORADA camera and iTEM 1233 software (Olympus Soft Imaging Solutions, GmbH, Münster, Germany).

### 4.5. Statistical Analysis

The mRNA expression levels (RQs), blood glucose levels, and animal weight are presented as the means ± SEMs. Data, depending on experimental design, were analyzed using Student’s *t* test or analysis of variance (one-way ANOVA) followed by Tukey’s post hoc test for multiple comparisons. The statistical analyses were performed using GraphPad Prism 6 (GraphPad Software, San Diego, CA, USA). Statistical significance was accepted at *p* < 0.05.

## 5. Conclusions

T2DM imposes a heavy health burden worldwide, and urgently requires new pharmacological strategies to prevent its long-term complications. Since bioactive sphingolipids are involved in regulating insulin signaling (through their action on cell survival and inflammatory response), using potential modulators of their expression and activity may provide a new therapeutic target against diabetes pathology. Based on our research, the use of metformin successfully reversed gene expression changes in sphingosine kinase 2 and S1P receptor 1 as well as the following genes encoding pro-inflammatory cytokines: IL-6 and TNF-α. Moreover, it improved the changes in the ultrastructure of the brain. These findings expand our knowledge on bioactive sphingolipid signaling and highlight the modulatory role of metformin in T2DM. However, a better understanding of the role and mechanisms by which S1P and metformin affect the changes that are present in the diabetic brain requires further elucidation on other models of the disease and brain structures. Additionally, it is essential to investigate the therapeutic window to correct the pathological changes that occur before and during the onset of T2DM.

## Data Availability

Dataset available on request from the authors.

## Supplementary Materials

The following supporting information can be downloaded at https://www.mdpi.com/article/10.3390/ijms26199832/s1.

## Abbreviations

The following abbreviations are used in this manuscript:

BS	bioactive sphingolipids
CNS	central nervous system
HFD	high-fat diet
MET	metformin
S1P	sphingosine-1-phosphate
S1PRs	sphingosine-1-phosphate receptors
SD	standard chow diet
Sph	sphingosine
SPHKs	sphingosine kinases
STZ	streptozotocin
T2DM	Type 2 diabetes mellitus
