# Supplementary material for: Metformin Induces Changes in Sphingosine-1-Phosphate-Related Signaling in Diabetic Mice Brain
Source: Int J Mol Sci. 2025 Oct 9;26(19):9832. doi: 10.3390/ijms26199832 (PMC12525125; doi:10.3390/ijms26199832)
Supplement: Supplementary file 1 [file ijms-26-09832-s001.zip › ijms-3809378-supplementary.pdf]

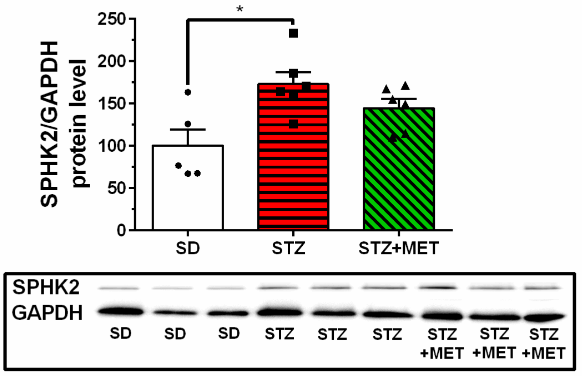

(a)

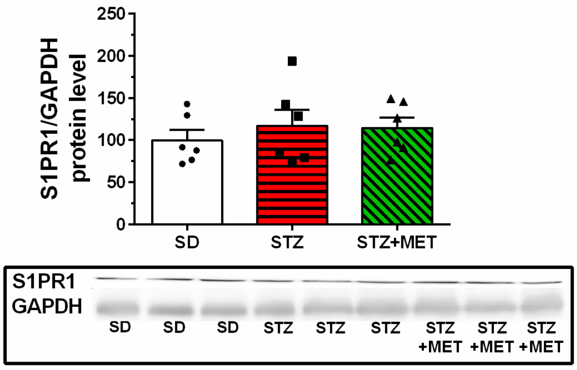

(b)

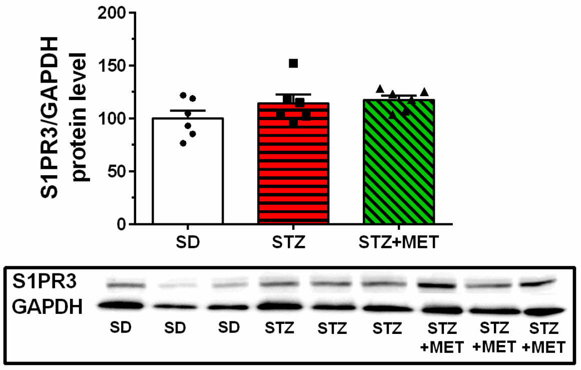

(c)

Supplementary figure S1. Changes in the protein levels of sphingosine kinase 2 (a) and receptors of sphingosine-1-phosphate (S1PR1 (b) and S1PR3 (c)) measured western blot in the brain hippocampus of control animals on standard diet (SD), animals received high-fat diet and streptozotocin (STZ) and mice simultaneously treated with metformin (STZ+MET). * $p<0.05$; as compared to the appropriate controls (5-6 animals for each group); ANOVA with Tukey post-hoc test.
